# Molecular and Cellular Mechanism of Leukemogenesis of ATL: Emergent Evidence of a Significant Role for HBZ in HTLV-1-Induced Pathogenesis

**DOI:** 10.1155/2012/213653

**Published:** 2011-11-24

**Authors:** Yorifumi Satou, Masao Matsuoka

**Affiliations:** ^1^Immunology Section, Division of Infectious Diseases, Imperial College, Wright-Fleming Institute, Norfolk Place, London W2 1PG, UK; ^2^Laboratory for Virus control, Institute for Virus Research, Kyoto University, Kyoto 606-8507, Japan

## Abstract

Adult T-cell leukemia (ATL) is a leukemia derived from mature CD4^+^ T cells and induced by human T-cell leukemia virus type 1 (HTLV-1) infection. Previous studies have revealed many possible molecular and cellular mechanisms of HTLV-1-induced leukemogenesis, but it still remains unknown how HTLV-1 transforms peripheral CD4 T cells in infected individuals. Given the fact that only 2–5% of infected individuals develop ATL, HTLV-1 infection alone is not sufficient for the transformation of infected cells. Host genetic and epigenetic abnormalities and host immunological status should be considered in attempting to understand the mechanism of the oncogenesis of ATL. Nonetheless, it is obvious that HTLV-1 infection dramatically increases the risk of leukemia generation from peripheral CD4 T-cells, in which the incidence of leukemia is quite low. Furthermore, the evidence that all ATL cases retain the HTLV-1 provirus, especially the 3′ region, indicates that HTLV-1-encoded genes play a critical role in leukemogenesis. Since increasing evidence indicates that the *HTLV-1 bZIP factor (HBZ)* gene plays a significant role in the pathogenesis of HTLV-1, we will discuss the cellular and molecular mechanism of ATL generation from the virological point of view, particularly focusing on HBZ.

## 1. Introduction

 Human T-cell leukemia virus type 1 (HTLV-1) is a complex retrovirus that infects approximately 10 to 20 million people worldwide [1]. In the late 1970s, adult T-cell leukemia (ATL) was identified as a distinct clinical entity based on its clinical and geographical features, suggesting an association with unknown infectious agents [2]. Thereafter, HTLV-1 was identified in a cell line derived from a patient with cutaneous T-cell leukemia in 1980 [3]. HTLV-1 has been shown to immortalize human T-lymphocytes *in vitro *[4]. In addition, HTLV-1 infection also induces chronic inflammatory diseases, such as HTLV-1-associated myelopathy/tropical spastic paraparesis (HAM/TSP) [5, 6], HTLV-1-associated uveitis [7], and HTLV-1-associated lung diseases [8]. The entire HTLV-1 sequence was determined [9] and various approaches were used to elucidate the pathogenesis of the virus. However, over 30 years after the discovery of HTLV-I, it is still not fully understood how HTLV-1 transforms mature CD4 T cells. Recent studies have provided emerging evidence of the significance of HBZ in HTLV-1 pathogenesis. In this review, we discuss the present understanding of HTLV-I infection from the virological aspect particularly via focusing on the role of the HBZ gene.

## 2. The Strategy of Replication in HTLV-1

**Figure 1:**
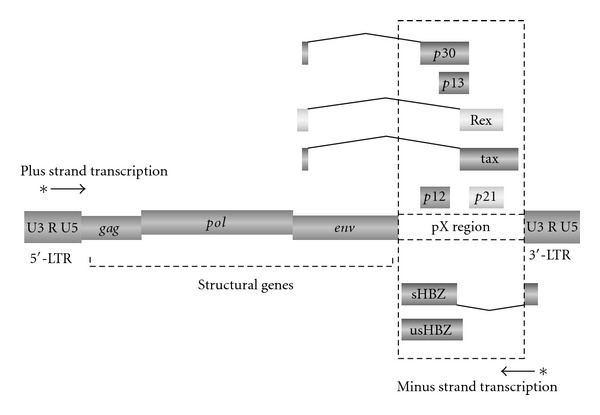
The structure of HTLV-1. HTLV-1 encodes accessory and regulatory genes in the pX region as well as viral structural genes.

 Once a retrovirus enters into host cells, the RNA genome is reverse-transcribed into a double-stranded DNA form, which is then integrated into the host chromosomal DNA by the viral integrase. The integrated viral DNA expresses viral genes to produce infectious virions. There are two retroviral replication patterns: *de novo* infection from infected cells to uninfected cells and clonal expansion of infected host cells [10]. Both free viral particle- and cell-to-cell-mediated *de novo* infection require expression of viral structural proteins and assembly of the viral particle. The expression of Tax enhances the transcription of viral structural genes from the plus strand of HTLV-1 in this situation (Figure 1). HTLV-1 has been reported to spread not via free viral particles but via cell-to-cell transmission through the virological synapse [11]. A recent study showed that the biofilm-like extracellular structure of infected cells plays a role in this cell-to-cell transmission of HTLV-1 [12]. This type of viral spread is thought to contribute to initial establishment of a population of infected cells. However the cells expressing Tax could be eliminated by the host CTL (cytotoxic T lymphocyte) response after establishment of host immunity against HTLV-1. After the establishment of anti-HTLV-1 immunity, HTLV-1 replicates predominantly using the second form of replication, clonal expansion of infected cells [13, 14]. In order to escape from the host immunity, HTLV-1 replicates as a provirus by increasing the number of infected host cells. In this phase, survival of HBZ-expressing cells is enhanced by the low immunogenicity of HBZ [15], which could be explained at least in part by the weak binding activity of HBZ peptide to MHC molecules [16]. 

 In the chronic phase of HTLV-1 infection, proviral load becomes stable in most infected individuals; yet there is a broad range of variation of proviral load among infected individuals. Since the variation of HTLV-1 sequence among infected individuals is very limited, host genetic factors including MHC class I molecules are thought to be important determinants of proviral load. Previously Tax has been considered as the most important antigen for the host immune response that controls proviral load [17], but recent evidence concerning lately identified viral protein HBZ has shifted the focus of research. The finding has suggested that individuals who possess MHC alleles which can efficiently bind and present peptides from HBZ have significantly lower proviral load, and are less likely to develop HAM/TSP [16], suggesting that HBZ expression is a critical determinant of viral persistence in chronic phase of HTLV-1 infection. Consistent with this idea, HBZ expression is constitutively detectable whereas Tax expression is frequently suppressed or diminished in ATL cells [18], which could be considered as the most highly expanded clone among many different HTLV-1-infected clones within an infected individual [19]. Even though HBZ might play an important role in viral persistence, *in vivo* persistence of HTLV-1 is decreased by mutation of other accessory genes, such as *p12*, *p13*, and *p30* [20–25], indicating that viral replication and proliferation of infected cells is controlled by these regulatory and accessory genes in harmony. 

 In summary, previous findings seem to be consistent with the theory that the initial expansion of HTLV-1 infection is due to* de novo* infection, which is driven by Tax, transactivator of HTLV-1 5′LTR. Once the host immune response to HTLV-1 has been established, HTLV-1 propagates in the host mainly by clonal expansion of infected cells via expressing HBZ, a viral antigen with low immunogenicity, as described previously [15].

## 3. The Host Cell of HTLV-1

 HTLV-1 has the potential to infect various cell-types such as T cells, B cells, macrophages, and dendritic cells (DCs) [26], but HTLV-1 can induce clonal expansion and transformation almost exclusively in CD4 T cells [10]. The mechanism underlying why HTLV-1 expands and transforms CD4 T-cell population needs to be uncovered. CD4 T-cells are generally partitioned into two subsets, effector T cells and regulatory T cells. The former plays crucial role in immune response by secreting cytokines that promote and activate immune systems, whereas the latter has been considered to suppress excessive immune responses to maintain the homeostasis of the immune system. Since the differentiation, function, and homeostasis are quite different between these two T-cell subsets, it is of great importance to consider the characteristics of each subset in order to understand how HTLV-1 utilizes and affects these CD4 T-cell subsets. 

### 3.1. HTLV-1 Infection in Effector CD4 T Cells

 To exert the function as effector T cells, naive T cells need to encounter their antigens, be activated, and be converted into effector/memory T cells. Previous reports have demonstrated that HTLV-1 infection is more frequently detected in effector/memory CD4 T cells than in naïve CD4 T cells [27, 28]. There is no compelling evidence to explain this tendency. We would propose three possible explanations as follows.

**(i) High Susceptibility to De Novo Infection**

* De novo* infection of HTLV-1 is achieved mainly by cell-to-cell infection, which is initiated by LFA-1-ICAM-1 interaction between infected cell and uninfected cells [29]. Since the expression level of LFA-1 and ICAM-1 in effector/memory CD4 T-cells is higher than that in naïve CD4 T cells [30], effector/memory CD4 T cells are likely to be more susceptible to *de novo* cell-to-cell infection than naïve CD4 T cells.

**(ii) High Proliferative Capacity**

Effector/memory CD4 T cells proliferate faster than naïve CD4 T cells *in vivo*. *In vivo* labeling of lymphocyte using deuterium-labeled glucose has shown that the doubling time of effector/memory CD4 T cells is 28 days, which is much shorter than the doubling time of naïve CD4 T cells, 199 days [31]. Long-term survival, a hallmark of memory CD4 T cell clones, also could contribute to the maintenance of HTLV-1* in vivo*. Therefore HTLV-1 infection in effector/memory CD4 T-cells is beneficial to clonal expansion of infected cells. Furthermore, HTLV-1-infected effector/memory CD4 T cells are reported to proliferate significantly faster than uninfected cells *in vivo* in HTLV-1 infected individuals [32].

**(iii) Enhancement of the Differentiation from Naïve to Effector/Memory CD4 T Cells**

Little is known about the impact of HTLV-1 infection on CD4 T-cell differentiation, because few studies have been focused on the effect of viral gene expression on T-cell differentiation to date. We have recently reported that the proportion of effector/memory CD4 T cells was increased in HBZ-transgenic (HBZ-Tg) mice [33], suggesting that HBZ expression can drive the differentiation from naïve T cells to effector T cells or enhance cell proliferation more strongly in effector/memory T cells than naïve T cells.

HTLV-1 utilizes this effector CD4 T-cell population as a host cell. It follows that this may induce the dysregulation of helper and effector function, contributing to the viral persistence.

### 3.2. HTLV-1 Infection of Regulatory CD4 T Cells

 CD4^+^CD25^+^FoxP3^+^ regulatory T cells (Tregs) have been identified as one of the major immunoregulatory mechanisms which prevent autoimmune disease [34]. Tregs are also involved in the downregulation of specific immune responses during infectious diseases. It has been reported that the frequency of Tregs is elevated in chronic viral infection, such as hepatitis C virus (HCV) [35]. The increased frequency of Tregs may help to prevent immune pathology but on the other hand may facilitate viral persistence. Indeed, the frequency of CD4^+^FoxP3^+^Tax^−^ T cells is inversely correlated with HTLV-1 specific CTL response, which could explain the variation of CTL response among infected people [36]. In addition to this general role of Tregs in chronic viral infection, there is a unique role of Tregs in HTLV-1 infection due to direct infection of CD4 T cells by HTLV-1, which include both progenitor Treg cells and Treg cells. The frequency of HTLV-1 infection in CD4^+^FoxP3^+^ cells is higher than other T-cell population [36], which we suggest maybe due to the following.

**(i) High Susceptibility to De Novo Infection**

Treg cells are known to contact with DCs frequently [37], which could increase the chance of *de novo* infection between DCs and Tregs. DCs are susceptible to HTLV-1 infection, and HTLV-1 infected DCs stimulate proliferation of T cells [38, 39]. A recent study also demonstrated that cell-free HTLV-1 efficiently infects DCs, and the infected DCs promote de novo infection of CD4 T cells [40].

**(ii) High Proliferative Capacity**

* In vivo* labeling of lymphocytes using deuterium-labeled glucose has shown that the FoxP3^+^ Tregs were extremely proliferative *in vivo* with a doubling time of 8 days [41]. HTLV-1 infection could further enhance proliferative activity of Tregs via expression of HBZ, which has been shown in Tregs of HBZ-Tg mice [33].

**(iii) Enhancement of the Differentiation of Tregs by HTLV-1 Infection**

HBZ enhances the generation of CD4^+^FoxP3^+^ T cells in transgenic mice, suggesting that HBZ has enhancing effect on generation and/or expansion of Foxp3^+^ Treg cells. As a mechanism, HBZ promotes the generation of FoxP3^+^ Tregs via enhancing the TGF-*β* signaling pathways [42], which is a crucial pathway for generation of induced Tregs.

**(iv) Advantage of Escape from the Host Immune Systems**

Tregs have an immune suppressive effect through both cell-contact-dependent and independent mechanisms [34]. Thus HTLV-1-infected Tregs should be more resistant to HTLV-1-specific CTL killing than HTLV-1-infected non-Tregs, resulting in preferential survival of HTLV-1-infected Tregs *in vivo*.

It seems reasonable that ATL cells would be stochastically derived from FoxP3^+^Tregs because of the high frequency of HTLV-1 infection in FoxP3^+^ Tregs. But it still remains unclear whether or not ATL is leukemia of FoxP3^+^ Tregs. Some studies have reported that ATL cells have regulatory function [43, 44], whereas other studies reported no regulatory function in ATL [45, 46]. Given the fact that the detection of FoxP3 expression in ATL cells is variable [47, 48], ATL is likely to be derived from both Treg cells and non-Treg cells. But the situation is more complicated because recent studies have indicated there is a plasticity between Treg cells and non-Treg cells [49]. Even when ATL cells do not express FoxP3, we cannot exclude the possibility that ATL cells were derived from FoxP3^+^ Tregs which have lost the FoxP3 expression during the process of leukemogenesis. Conversely, even when ATL cells do express FoxP3, we cannot exclude the possibility that FoxP3 expression is aberrantly induced in non-Tregs by HTLV-1 infection. Furthermore, even when regulatory function of FoxP3^+^ ATL cells is not evident, there is a possibility that HBZ expression inhibits the function of FoxP3 and impairs the function of the host Tregs [33].

Taken together it is difficult to make a clear conclusion about the relationship between HTLV-1 and the CD4 T-cell subset of host cells at present, but we need to continue the effort to reconcile the complexity to understand the pathogenesis of HTLV-1.

## 4. The Minus Strand Viral Gene: HBZ

 The presence of a transcript from the minus strand of the HTLV-1 provirus has been reported in 1989 [50], but HBZ has not been described until recently. HBZ was identified using the CREB-2 binding protein in the yeast two-hybrid screening system using HTLV-1 infected MT-2 cells [51]. HBZ suppresses Tax-mediated viral gene expression from the 5′LTR by interacting with CREB-2. Therefore, HBZ is a negative regulator for viral gene expression. The following data describing expression of HBZ in primary HTLV-1-infected cells have highlighted the significant role of HBZ in the pathogenesis of HTLV-1 [18, 52–54]. 

### 4.1. Minus Strand Transcription of HTLV-1

#### 4.1.1. Transcriptional Pattern of HBZ

 There are two major transcripts of HBZ, spliced and unspliced HBZ (Figure 1) [18, 55, 56]. Both HBZ transcripts have been detected in ATL cells [56]. Spliced HBZ mRNA expression is correlated with disease severity of HAM/TSP [53]. The level of HBZ expression is higher than that of tax in uncultured primary cells [52, 53]. A recent study has proposed one possible reason why immunogenicity of HBZ is low. They reported that HBZ-RNA is preferentially retained in the nucleus, which may result in the low translation efficiency of HBZ and contribute to its immune escape [57]. It is of great interest to elucidate the mechanism why HBZ-RNA is retained in the nucleus because that also could also explain how HBZ RNA induces a growth-promoting effect on T cells [18].

#### 4.1.2. Possible Effect of Integration Site on HBZ Transcription

 It is possible to evaluate the expression level of HBZ in total PBMC at population level but not at single clone level at present. There are many different clones even within one infected individual [19]. Every infected clone has its own unique integration site; so the difference in the integration site may affect the expression of HBZ; for example, the epigenetic features of HTLV-1 provirus are determined in part by surrounding host genomic features. When HTLV-1 integrated in heterochromatic region of the host genome, the transcriptional machinery has difficulty accessing the 3′LTR promoter region of HBZ (Figure 1) [58], which could inhibit transcription of HBZ. A previous study has indeed shown that integration sites in ATL cells are frequently located within transcriptional units of host genes but rarely located in a heterochromatic region of human genome compared with those in untransformed infected cells [59]. This result suggests that an HTLV-1-infected clone that carries a provirus integrated within a transcriptionally active region is susceptible to ATL generation. A novel powerful method of integration site analysis using a high-throughput technique has been developed recently and provided more detailed and precise information on the integration site of HTLV-1 [19]. In order to elucidate the effect of the integration site on the clonal dominance, further experiments will be required to investigate the underlying molecular mechanism of the transcriptional interaction between HTLV-1 provirus and the surrounding host genome.

### 4.2. Molecular Property of HBZ Protein

**Figure 2:**
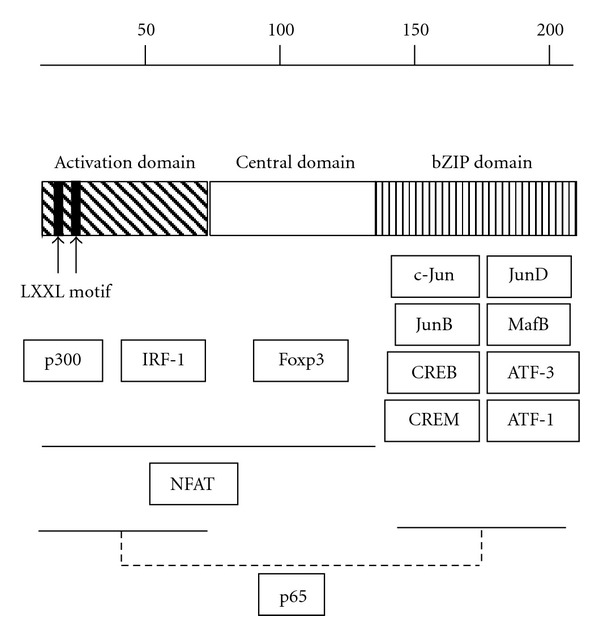
The structure of HBZ and interacting host factors. HBZ could play a crucial role in the HTLV-1 pathogenesis by interacting cellular factor as shown in this figure.

 HBZ has been reported to interact with various host factors via its three distinct coiled-coil domains, including activation domain, central domain, and bZIP domain (Figure 2). Transcriptional coactivator p300 and interferon regulatory factor IRF-1 interact with the activation domain of HBZ [60, 61]. HBZ induces the activation of TGF-*β* signaling pathway by forming a complex with p300 and Smad proteins [42]. HBZ also activates Dkk1 expression through its KIX domain that interacts with p300 [60, 62]. The central domain of HBZ is responsible for the interaction with Foxp3 [33], which results in the dysfunction of transcriptional activity of Foxp3. The bZIP domain of HBZ is responsible for the interaction with the host bZIP factors, such as c-Jun, JunB, JunD [63, 64], CREB, CREM, ATF-1 [65], ATF-3 [66], and MafB [67]. These data have demonstrated that HBZ can form complexes with host factors, resulting in the dysregulation of the host cell-signaling pathways. Although it is very difficult to identify how each molecular mechanism affects the fate of infected cells, it could contribute to the phenotype of infected cells in a coordinated manner. It is striking that HBZ interacts with transcription factors that play a critical role in CD4 T cells such as AP-1, NF-*κ*B [68], and FoxP3; therefore these molecular interactions could explain the observed cell-type specificity of transformation induced by HTLV-1.

### 4.3. Effect of HBZ Expression on Viral Persistence and HTLV-1-Related Pathogenesis

Experiments using a molecular clone of HTLV-1 deleted for HBZ demonstrate that HBZ is dispensable for the HTLV-1-mediated T-cell transformation *in vitro*. However HBZ plays an indispensable role in persistent viral infection *in vivo *[69]. More recently, in the macaque model of HTLV-1 infection, reversion of HBZ knock-out HTLV-1 to wild-type HTLV-1 was observed within weeks from infection, also indicating that HBZ plays a crucial role in the persistent infection of HTLV-1 [24]. Tax is thought to be responsible for the *in vitro* transformation induced by HTLV-1 infection, in which Tax is allowed to express because of the absence of selection pressure by the host immune systems. Also, since Tax has a strong capability to induce genomic instability in infected cells [70–73], Tax could contribute to the accumulation of host genomic abnormalities related to oncogenesis, even at its limited expression level *in vivo.* Nonetheless, Tax-expressing cells would be susceptible to elimination by CTL in an immune competent host. In contrast, the immune selective pressure on HBZ may be significantly lower allowing expression of HBZ mRNA *in vivo* [15, 16], which should contribute to the persistence of HTLV-1. More surprisingly, the phenotype of HBZ-Tg mice has demonstrated that HBZ expression in CD4 T cells *in vivo* could induce the disease phenotype of HTLV-1 infection such as chronic inflammation and T-cell lymphoma [33]. Thus, the transgenic expression of either Tax or HBZ induces both T-cell lymphoma and chronic inflammation [33, 74–76]. It is of great interest how Tax and HBZ synergistically, competitively, or independently contribute to various aspects of the viral pathogenesis. For example, Tax activates the NF-*κ*B signaling pathway, but HBZ represses the canonical pathway of NF-*κ*B [42]. A recent study has shown that HBZ can alleviate cellular senescence induced by Tax-mediated NF-*κ*B hyper-activation [77]. Future experiments should aim to clarify the role of Tax and HBZ in each aspect of HTLV-1-associated pathogenesis.

## 5. Future Direction of Treatment

 Since the identification of ATL as a distinct clinical entity, some progress has been made in preventing and treating the disease. In particular, the identification of a transmission route from the mother to her child through breast milk enables us to reduce *de novo* HTLV-1 infection [78]. However, an effective therapeutic strategy for ATL remains elusive. In particular, there are few available therapies that target HTLV-1 to date. Some reports have shown the efficiency of combination therapy of zidovudine and interferon alpha (AZT/IFN) for treatment of ATL [79], even though little is known about underlying mechanism of AZT/IFN therapy. Recent approaches using allogeneic bone marrow transplantation have significantly improved the prognosis of ATL patients [80, 81], suggesting that enhancement of the immune response to HTLV-1 is a possible strategy for treatment of HTLV-1-associated human diseases [82]. As we discussed in this review, if we could activate viral gene expression, we could remove the infected cells by recruiting HTLV-1-specific CTLs. Several preclinical and clinical studies using valproate, a histone deacetylase (HDAC) inhibitor, have already been performed and shown the efficacy of this therapeutic approach. Histone modification is reported to contribute to the proviral gene expression of HTLV-1 [83–86]. Treatment with valproate enhances the expression of Tax, and the resulting exposure to anti-Tax CTL may explain the observed reduction in HTLV-1 proviral load [87, 88]. The combination of HDAC inhibitor and antiretroviral drugs remarkably reduced the proviral load in STLV-1 naturally infected baboons [89]. In order to find more efficient and specific molecular therapeutic targets, it is necessary for us to clarify the nature of leukemogenesis induced by HTLV-1.
